# Phosphorus application improves grain yield in low phytic acid maize synthetic populations

**DOI:** 10.1016/j.heliyon.2021.e07912

**Published:** 2021-09-02

**Authors:** Mohammed A.E. Bakhite, Nkanyiso J. Sithole, Lembe S. Magwaza, Alfred O. Odindo, Shirly T. Magwaza, Khayelihle Ncama

**Affiliations:** a Discipline of Crop Science, School of Agricultural, Earth and Environmental Sciences, University of KwaZulu-Natal, Private Bag X01, Scottsville, 3209, Pietermaritzburg, South Africa; b Crop Science Department, Faculty of Natural and Agricultural Science, North-West University Private Bag X 2046, Mmabatho 2035, South Africa; c Food Security and Safety Niche Area, Faculty of Natural and Agricultural Sciences, North-West University, Mmabatho, South Africa; d Discipline of Horticultural Science, School of Agricultural, Earth and Environmental Sciences, University of KwaZulu-Natal, Private Bag X01, Scottsville, 3209, Pietermaritzburg, South Africa; e Department of Agricultural Science, University of Zululand, Private Bag X1001, KwaDlangezwa 3886, South Africa

**Keywords:** Low phytic acid maize, *Zea mays*, High phytic maize, Maize seed quality

## Abstract

Maize mutants with low phytic acid have a compromised overall agronomic performance that results in low yields. This study was conducted to investigate the effect of P (18, 26 and 34 mg/kg) on the agronomic performance of low and high phytic acid (LPA and HPA) maize synthetic populations of tropical origin, compared to two commercial hybrids (SC701 and LS8520). Subsequently, a germination test was performed on the seeds produced from the different levels of P fertilizer application rates. The germination test was conducted in the laboratory, using a germination paper towel, while the agronomic study was conducted in a controlled environment. The measured parameters included days to 50% flowering, plant height, and grain yield, as well as the final germination and germination velocity index. The results found that the grain yield increased by 1.30, 0.51, 2.41 and 1.87 t/ha in LPA, HPA, SC701 and LS8520, from the application of 18–26 mg/kg of P, respectively. However, there were non-significant differences (p > 0.05) in the grain yields of all varieties at a P application of 26 and 34 mg/kg. The final germination increased by 4% and 2% in LPA and LS8520, respectively, with the increase in the P application rate being from 18 to 26 mg/kg. However, no significant differences (p > 0.05) were found in the final germination percentage of all varieties at 26 mg/kg of P. This study indicated that the optimum application of P at planting enhances the overall performance of the LPA maize synthetic population to a level that is comparable to commercially-grown varieties.

## 1 Introduction

Phytic acid (*myo*-inositol 1,2,3,4,5,6-hexakisphosphate or Ins P_6_) also known as phytate or phytin is the main storage form of phosphorus in maize seeds that has a vital nutritional role for humans and livestocks (Lehrfeld, 1994; Raboy, 2001). Phytic acid can be extracted from mature seeds as a mixture of *myo*-inositol hexakisphosphoric acid salts (Modi and Asanzi, 2008). This highly negatively charged chemical has a strong binding affinity to cations such as Fe^2+^, Zn^2+^, K+, Ca^2+^, and Mg^2+^ (Iwai et al., 2012). Phytin accumulate during the seed development and maturation phase, which is the period of reserve synthesis and accumulation (Lott et al., 1995). As a result, mature seeds contain large amounts of phosphorus in the form of phytic acid, which supports growth during the early stages of seed germination and establisment (Mandizvo and Odindo, 2019). Phytic is catabolized by the hydrolysis reaction during seed germination to release inorganic phosphates, inositol, and various minerals from the phytate (Bohn et al., 2007).

A vital role of phytic acid in seed physiology is that of protection against oxidative stress during the seed's lifespan. Seeds are affected by oxidative stress during the last phase of maturation when the seed tissue undergoes desiccation (Bailly 2004). Badone et al. (2010) endowed the antioxidant properties of phytic acid against reactive oxygen species in maize (*Zea mays*) seeds. This was in agreement with the gradual decrease noticed by Doria et al. (2009) in antioxidant reserves during seed storage and ageing. Therefore, phytic acid has a major role in maintaining the quality of the seeds by protecting the viability of the embryo.

Although phytic acid has such a vital role in seed physiology, in humans and livestock which consumes them, it binds important mineral cations, such as Zn^2+^, Fe^2+^, Ca^2+^ Mg^2+^ and K^+^, making them unavailable (Wu et al., 2009; Azeke et al., 2011). Monogastric animals, including humans, lack digestive enzymes called phytases in their digestive tract and cannot process the phytates that are present in seeds (Sparvoli and Cominelli, 2015). As a result, phytic acid is poorly digested and decreases the nutritional value of the seeds by limiting the mineral cations bioavailability. Poor mineral bioavailability is hypothesized to be the one of causes of mineral deficiencies in populations which depends on maize as a staple crop (Schlemmer et al., 2009; Sparvoli and Cominelli 2015; Garcia-Oliveira et al., 2018).

In recent years, efforts has been made to improve the nutritional quality of maize seeds by reducing the phytic acid content. Low phytic acid mutants (*lpa 1-1*) of cereal and legumes crops have been isolated, genetically mapped and developed (Shunmugam et al., 2014; Yatou et al., 2018). Cominelli et al. (2020) reported that these low phytic acid mutations have the potential to alleviate the nutritional problems caused by phytic acid, in both human and animal consumers. Therefore, low phytic acid maize may provide better nutrition for human populations that depend on grains as their staple food, particularly in sub-Saharan Africa (SSA) (Shunmugam et al., 2014; Yatou et al., 2018).

Reducing the phosphorus content in the seeds to improve their nutritional quality may be not practical, since it reduces the seed quality and yield. Preliminary investigations into the *lpa* maize mutants, of temperate origin, have shown that the seed quality, performance and yield is reduced, when compared to normally-grown or improved cultivars (Modi and Asanzi, 2008; Pilu et al., 2005; Naidoo et al., 2012). This is because phytic acid, and its synthetic pathways are central to a number of metabolic, developmental and signalling pathways that are important to plant function and productivity; thus, reducing phytic acid can translate into low yields or stress susceptibility (Raboy, 2009). Hence, phosphorus nutrition during planting can be associated with improving the overall performance and yield in *lpa* mutants. However, comparative studies on soybean seeds have found that reducing phytic acid did not have an effect on seed germination in seeds with a high (1.0 mg/seed), medium (0.59 mg/seed) and low (0.19 mg/seed) PA concentration. This implied that these seeds contain P reserves that are far above the level that is needed for seed germination and early seedling growth. Therefore, to overcome the problem of malnutrition in maize, it is important to screen and select the population of low phytic acid with P reserves that are adequate for seed germination and early seedling growth. In addition, although *lpa* genes have been reported in maize cultivars from temperate regions (Raboy, 2000), no studies have been conducted on maize of tropical origin.

Therefore, the objective of this study was to investigate the response to phosphorus application of low and high phytic acid maize populations of tropical origin. Both the low and high phytic synthetic populations were derived from the same tropical population and were selected on the basis of their phytic acid content. Consequently, the seed quality, with respect to the germination and vigour of the seeds produced from different fertilizer application rates, was evaluated.

## 2 Materials and methods

### 2.1 Plant material

Low Phytic Acid (LPA) and High Phytic Acid (HPA) seeds were sourced from the African Center for Crop Improvement (ACCI). Both the LPA and HPA seeds were F2 generation breeding populations that were selected, based on their phytic acid content. The 24 lines with LPA, ranging from 1.27 to 32 mg/g, were allowed to randomly pollinate each other for two generations to form the LPA synthetic population. The other 51 lines, from the same tropical F2 population with HPA, ranging from 43 to 113 mg/g, were allowed to cross-pollinate each other to form the HPA synthetic population. The SC701 and LS8520R (484) hybrid seeds that are coded as LS8520 in this study were sourced from a local seed company, McDonald Seeds. The seeds used in this study were produced in the same growing season and under identical environmental conditions.

### 2.2 Phytic acid analysis assay

Phytic acid content of the varieties was determined following a method by Aina et al. (2012). The test was conducted by preparing 25 samples from each variety. For each sample, 2 g of flour was weighed and put into a 250 ml conical flask. The sample was soaked for 3 h in 100 ml of 2% concentrated HCl before it was filtered through a No.1 Whatman filter paper. The 100 ml of filtrate and 10 ml of distilled water was added, to adjust the acidity. An amount of 10 ml of a 0.3% ammonium thiocyanate solution was added to the solution as an indicator, before the solution was titrated with a standard iron II chloride solution containing 0.00195 g iron/ml, till the endpoint was reached when a yellow colour was observed and persisted for 5 min. The percentage of phytic acid was calculated by using Eq. (1).

(1) Phytic acid (mg/g) = y × 1.19 × 100

Where: y = titre value × 0.00195 g

### 2.3 Controlled environmental conditions

The experiment was conducted in a greenhouse at the University of KwaZulu-Natal, Pietermaritzburg, South Africa (29°35′S, 30°25′E). The average night and day temperatures were 28 °C and 32 °C, respectively, at a natural day length. The Relative Humidity (RH) was maintained at approximately 60% throughout the growing season. The temperature and RH were monitored by using a HOBO 2K logger (Onset Computer Corporation, Bourne, USA).

#### 2.3.1 Experimental design and trial management

The experiment was laid out as a Completely Randomized Design (CRD) with two treatment factors: the varieties (LPA & HPA and hybrid SC701 & LS8520) and the phosphorus (P) levels [18 mg/kg (residual, control), 26 mg/kg (optimum for maize production under selected soil) and 34 mg/kg (high)], replicated three times (Modi and Asanzi, 2008). The phosphorus levels, derived from superphosphate, were applied immediately before planting in 25 L pots containing 20 kg of soil. Two plants were grown in each pot filled with soil (clay loam) collected from Ukulinga Research Farm and, soon after emergence, this was reduced to one plant. The fertility status of this soil is presented in Table 1 and it was fertilized based on the recommendations for optimum maize production.

**Table 1:** Physical and chemical properties of soil used in the controlled environmental facility.

Soil characteristic	Quantity
Sample density (g/ml)	1.11
N (%)	0.19
P (mg/kg)	18.00
K (mg/kg)	185.50
Ca (mg/kg)	1281.00
Mg (mg/kg)	306.50
Exchangeable acidity (cmol/L)	0.07
Total cations (cmol/L)	9.46
Acid saturation (%)	1.00
pH (KCl)	4.63
Zn (mg/kg)	4.90
Mn (mg/kg)	72.00
Cu (mg/kg)	15.00
Organic carbon (%)	2.10
Clay (%)	35.00

#### 2.3.2 Measurement of growth parameters and yield

Three plants from each variety were randomly selected and the plant height was measured, using a tape measure. The height was measured from the soil to the base of the tassel. The number of leaves, with at least a 50% green area, was counted until flowering. The days to tasseling were recorded as the number of days from sowing, until 50% of the plant population had been tasseled. Finally, the yield components were measured at harvest, when the seeds are physiologically mature.

### 2.4 Seed quality tests

#### 2.4.1 Seed electrical conductivity

The electrical conductivity was assessed using a CM 100-2 single cell analyzer (Reid and Associates, Durban, South Africa). The mass of four replicates of 30 seeds per variety were measured, using a weighing balance, before being soaked in 2 ml wells filled with distilled water. The Electrical Conductivity (EC) of the seeds was recorded over a period of 24 h.

#### 2.4.2 Standard germination test

Seed germination test was determined using a completely randomized design following the method prescribed by ISTA (2012). Briefly, four replicates consisting 25 seeds per variety were placed between two layers of brown germinating towel. Paper towel were rolled and closed at both ends and put into plastic zip-lock bag. Thereafter, these were incubated in a germination chamber set at 25 ± 1 °C. Germination was monitored daily by counting seeds with a radicle protrusion of at least 2 mm. The final germination was determined on day 7 according to (AOSA, 1992) based on normal seedling. Following this, seed vigor characteristics of root length and shoot length were determined. The following germination indices were calculated:

• Mean Germination Time (MGT), Eq. (2) (Ellis and Roberts, 1981)

(2) $$\text{MGT ​}=\text{ ​}\frac{\sum \mathrm{Dn}}{\sum n}$$

where:

MGT = mean germination time,
*n* = the number of seeds which were germinated on day D, and
*D* = number of days counted from the beginning of germination.
• Germination Velocity Index (GVI), Eq. (3) (Maguire 1962)

(3) GVI = G1/N1 + G2/N2 +… + Gn/Nn

where:

GVI = germination velocity index
G1, G2…Gn = number of germinated seeds in first, second… last count.
N1, N2…Nn = number of sowing days at the first, second… last count.

#### 2.4.3 Accelerated ageing vigour test

The seeds were placed in a plastic box with temperature set at 41 °C and ≈95% Relative Humidity (RH) for 48 h, followed by a 72 h storage period, following the standards set by ISTA (2012). The unaged seeds (0 day) were used as the control. The ageing chamber was a plastic box (8 cm × 8 cm) with a lid, which was placed onto a wire tray with a 10 cm × 10 cm x 2 cm (length x width x depth) mesh screen, the pore size of which was 1.89 mm. In each accelerated ageing box, 40 g of sodium chloride and 100 ml of distilled water was added and a dry screen tray was inserted to prevent the splashing of water onto the screen. After each period of accelerated ageing treatment, the standard germination test was performed, as described in Section 2.4.2.

### 2.5 Statistical analysis

All data collected were subjected to the Analysis of Variance (ANOVA), using GenStat® 18^th^ Edition (VSN International, Hemel Hempstead, UK, 2011). The means were separated using Fischer's Least Significant Difference (LSD) at a 5% level of significance.

## 3 Results

Significant differences (p < 0.05) in phytic acid were observed between the varieties (Table 2). The HPA maize varieties recorded the highest (p < 0.05) phytic acid content, followed by LS8520 and SC701, while the LPA varieties recorded the lowest.

**Table 2:** Phytic acid content of different maize seeds of LPA seeds, compared to HPA, SC701 and LS8520.

Variety	Phytic acid (mg/g)
LPA	18.58^a^
HPA	70.73^d^
SC701	41.95^c^
LS8520	32.75^b^
CV (%)	28.00
LSD	6.45
*p*-value	<.001

Note: LPA = low phytic acid, HPA = high phytic acid, SC701 = white maize, and LS8520 = yellow maize. Values within the same column sharing the same letter are not significant different at p < 0.05.

### 3.1 Growth parameters and yield

Significant differences (p < 0.05) were observed in the plant height, which related to the fertilizer application rate. It was only at the residual level of P where the plant height of LPA varieties was significantly lower than that of the SC701 varieties (Table 3). No significant (p > 0.05) differences were found in the plant height between the optimum and high rates of P fertilizer application. However, the plant height was observed to increase with an increase in the P application rate from the residual to the optimum level. In contrast, the plant height did not increase (p > 0.05) from the optimum P fertilizer application rate to a higher level of the P fertilizer application rate.

**Table 3:** Growth and yield components of LPA seeds, compared to HPA, SC701 and LS8520 seeds under different P concentrations.

Variety	P (mg/kg)	Plant height (cm)	Leaf number	Days to Tassel	Days to Silking	Days to Anthesis	1000 Grain mass (g)	Yield t/ha
LPA	18	215^a^	15.2^ab^	65.0^b^	72.5^d^	71.5^d^	205^a^	1.70^a^
HPA		221^abc^	14.1^a^	64.3^b^	71.5^cd^	71.5^d^	257^cde^	2.4^ab^
SC701		225^bcd^	16.3^bc^	57.3^a^	67.7^bcd^	60.7^abc^	236^abc^	2.0^ab^
LS8520		219^ab^	16.0^bc^	57.2^a^	67.5^bcd^	61.3^abc^	214^ab^	2.03^ab^
LPA	26	233^e^	15.7^bc^	64.1^b^	66.9^bc^	66.4^cd^	233^abc^	3.0^abcd^
HPA		228^cde^	15.0^ab^	66.2^b^	64.3^b^	66.9.9^cd^	282^e^	2.91^abc^
SC701		231^de^	16.8^c^	56.1^a^	56.5^a^	57.5^ab^	248^cd^	4.41^cd^
LS8520		230^de^	16.1^bc^	57.0^a^	54.1^a^	57.3^ab^	237^bc^	3.9^cd^
LPA	34	228^cde^	15.5^abc^	66.1^b^	66.7^bc^	64.7^bcd^	275^de^	3.23^bcd^
HPA		234^e^	15.1^ab^	63.0^b^	65.0^b^	64.5^bcd^	342^f^	3.41^bcd^
SC701		231^de^	15.9^bc^	55.3^a^	54.2^b^	57.0^a^	285^e^	4.48^d^
LS8520		232^de^	16.2^bc^	54.8^a^	55.9^a^	56.1^a^	258^cde^	4.03^cd^
LSD_(v×p)_		4.51	0.88	2.06	3.03	4.26	17.75	0.39
*P*(v)		0.055	<.001	<.001	<.001	<.001	<.001	<.001
(P)		0.055	<.001	<.001	<.001	<.001	<.001	<.001
(Pxv)		<.001	0.087	0.057	<.001	<.001	<.001	<.001
CV		1.2	3.4	2.0	2.8	4.0	4.1	9.4

Note: LPA = low phytic acid, HPA = high phytic acid, SC701 = white maize and LS8520 = yellow maize. Values within the same column sharing the same letter are not significant different at p < 0.05.

The leaf numbers of the HPA varieties at the residual P level were significantly lower (p < 0.05) than those of other varieties. There were no significant differences in the leaf numbers of the other varieties (Table 3). The leaf number (16.8) of SC701 was significantly (p < 0.05) higher than all other varieties at the optimum P level. No significant differences (p > 0.05) were found between the other varieties: HPA (15.0), LPA (15.7) and LS8520 (16.1). No significant differences (p > 0.05) were found in the leaf numbers within the varieties at a high rate of P application.

The number of days to tasseling of commercial varieties (SC701 & LS8520) was significantly lower than those of the LPA and HPA varieties. On average, the days to tasseling of commercial varieties were 56 and those of the LPA and HPA varieties were 65. The days to silking and anthesis were influenced by the phosphorus treatments within each variety. The commercial varieties were found to take less time to silking and anthesis than the low and high phytic acid varieties. No significant differences (p > 0.05) were found in the grain yield at a residual P fertilizer application (Table 3). However, the yield was found to increase with the increase in the P fertilizer application rate. At the optimum P fertilizer application rate, no significant (p > 0.05) differences were found in the grain yield in all varieties. The increase in the P fertilizer application rate beyond the optimum application did not significantly (p > 0.05) increase the grain yield.

### 3.2 Standard germination test

There were no significant differences (p > 0.05) in the seed-lots of the LPA, HPA and SC701 varieties at a residual level of the P fertilizer application rate (Table 4). Non-significant differences (p > 0.05) were only found in LS8520, where Seed-lot 2 was observed to be higher than Seed-lot 3. At the optimum level of P application, no significant differences (p > 0.05) were found between the seed-lots of all varieties. At a high level of P application, non-significant differences were found between the seed-lots of different varieties. The final germination percentage was also observed to increase from the residual to the optimum P application rate.

**Table 4:** Final germination percentage of the LPA, HPA and commercial hybrids (SC701 and LS8520) seed-lots in response to the P application rate.

Lots	P (mg/kg)	Final germination (%)			
		LPA	HPA	SC701	LS8520
1	18	92.0^a^	96.0^ab^	100^c^	95.0^cd^
2		92.0^a^	96.0^ab^	100^c^	96.0^de^
3		93.0^ap^	94.8^ab^	100^c^	94.0^bc^
4		91.0^a^	96.0^ab^	100^c^	95.0^cd^
1	26	96.0^cd^	95.0^ab^	100^c^	97.1^ef^
2		95.0^bc^	96.0^ab^	100^c^	98.0^f^
3		96.0^cd^	95.0^ab^	100^c^	98.0^f^
4		97.0^cd^	94.0^a^	100^c^	97.0^ef^
1	34	97.0^cd^	95.0^ab^	99^ab^	94.0^bc^
2		95.0^bc^	97.0^b^	99.5^bc^	93.0^ab^
3		98^d^	95.1^ab^	98.0^a^	92^a^
4		97.2^cd^	97.0^b^	99.0^ab^	93.0^ab^
LSD_(lot×p)_		2.66	2.35	0.50	1.97
*P* (lot)		0.194	0.216	0.196	0.237
(P)		<.001	0.224	<.001	<.001
(Pxlot)		0.516	0.461	0.132	0.497
CV		2.0	1.7	0.4	1.4

Note: LPA = low phytic acid, HPA = high phytic acid, SC701 = white commercial maize variety and LS8520 = yellow commercial maize variety. Values within the same column sharing the same letter are not significant different at p < 0.05.

**Figure 1:**
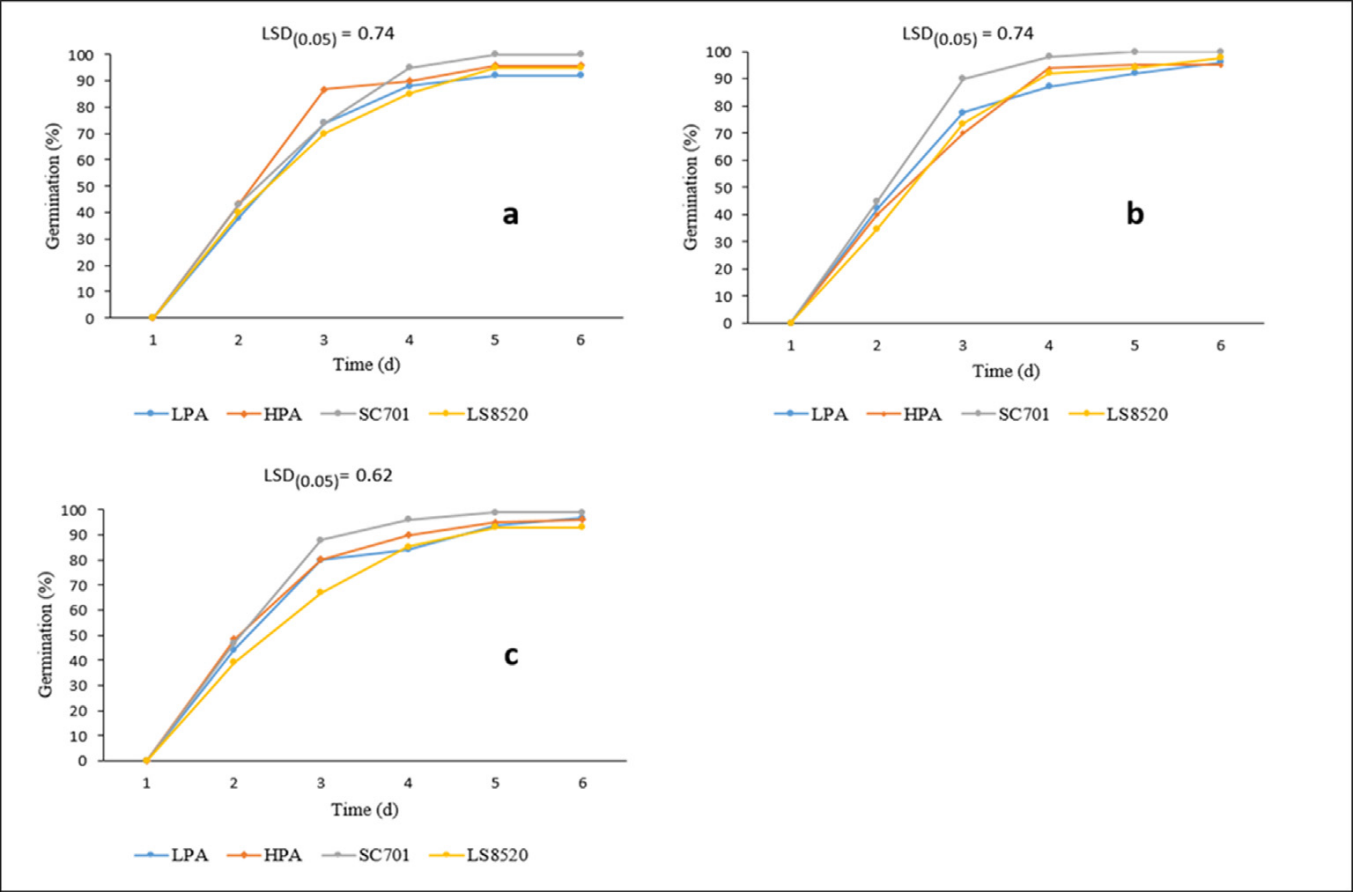
Daily germination in response to the P application rate of low phytic acid (LPA), compared to HPA (high phytic acid) and two commercial hybrids, SC701 (white maize) and LS8520 (yellow maize). Note: a = reisidual P level, b = optimum P level and c = high P level.

Significant differences (p < 0.05) were observed in the germination between the varieties grown with different levels of P (Figure 1). In all P application rates, the HPA and SC701 varieties showed a high and fast germination rate. The opposite was observed in the LS8520 variety, while LPA fluctuated. The increased rate of P application increased the germination rate for LPA, especially in the initial stages of germination. LPA maize showed a lower germination percentage than all the other varieties, at a residual P fertilizer application rate, while that of SC701 was the highest (Table 5). The increase in the P fertilizer application rate, from the residual to the optimum levels, enhanced the germination percentage of LPA by 4%. There were no significant responses (p < 0.05) among the varieties to the increase in P application from the optimum to the high P application rate. There were no significant differences (p < 0.05) observed in the MGT between the varieties in their response to P application (Table 5). The same trend was observed in the GVI (Table 5). The GVI of LPA increased (5% increase) compared to HPA, SC701 and LS8520, with the increasing phosphorus application, from the residual to the high P application rate (Table 5).

**Table 5:** Seed performance of LPA, compared to HPA and commercial hybrids (SC701 and LS8520), in response to the P application rate.

Varieties	P (mg/kg)	Final germination (%)	MGT	GVI	EC (μS g^−1^)	Root length	Shoot length	Root: shoot
LPA	18	92.0^a^	2.82^a^	35.3^a^	16.7^bc^	10.6^a^	7.62^a^	1.39^a^
HPA		95.7^abc^	2.74^a^	38.4^abc^	12.5^ab^	13.5^bcd^	9.15^abcd^	1.49^a^
SC701		100^c^	2.88^a^	38.2^abc^	13.2^abc^	12.8^bc^	8.80^abc^	1.45^a^
LS8520		95.0^abc^	2.79^a^	35.2^a^	17.67^c^	12.0^ab^	7.95^ab^	1.50^a^
LPA	26	96.0^abc^	2.81^a^	36.4^abc^	13.0^abc^	12.5^abc^	8.45^ab^	1.49^a^
HPA		95.0^abc^	2.61^a^	36.2^abc^	10.9^a^	14.1^cd^	11.0^de^	1.28^a^
SC701		100^c^	2.49^a^	39.2^bc^	10.4^a^	13.0^bc^	9.62^bcd^	1.36^a^
LS8520		97.7^bc^	2.99^a^	35.8^ab^	12.5^ab^	11.9^ab^	8.27^ab^	1.41^a^
LPA	34	96.8^abc^	2.84^a^	37.2^abc^	11.0^a^	12.8^bc^	9.70^bcd^	1.33^a^
HPA		96.0^abc^	2.74^a^	38.5^abc^	9.80^a^	15.5^d^	11.97^e^	1.29^a^
SC701		99.0^c^	2.66^a^	39.7^c^	9.00^a^	13.5^bcd^	10.7^cde^	1.26^a^
LS8520		93^ab^	2.96^a^	35.1^a^	10.50^a^	12.3^abc^	8.60^ab^	1.43^a^
LSD(G×P)		3.22	0.30	2.21	2.77	1.23	1.13	0.96
*P* (G)		<.001	0.035	<.001	0.001	<.001	<.001	0.330
(P)		0.172	0.506	0.268	<.001	<.001	<.001	0.034
(PxG)		0.031	0.278	0.263	0.371	0.128	0.207	0.038
CV		2.0	7.8	4.2	13.4	6.7	8.5	9.9

Note: LPA = low phytic acid, HPA = high phytic acid, SC701 = white commercial maize variery and LS8520 = yellow commercial maize variety. Values within the same column sharing the same letter are not significant different at p < 0.05.

The results showed significant differences (p < 0.05) in the electrical conductivity (EC (μS g^−1^) (Table 5). The differences in EC were only observed at the residual level of P application, where the LS8520 varieties had a significantly higher EC than the LPA and HPA varieties. No differences (p > 0.05) were found at the optimum P level application and the high P level application rates within the treatments. The increase in the P application rate had no influence on the EC. Significant differences (p < 0.05) were found in the root and shoot length (Table 5). At the residual level of P application rate, LPA maize had a significantly (p < 0.05) lower root length than the HPA and SC701 varieties (Table 5). At the optimum P application rate, the root length of LPA varieties improved significantly (p > 0.05), and it was similar (p > 0.05) to all other varieties. A further increase in the P application rate to the higher level caused no significant differences (p > 0.05) between the LPA, SC701 and LS8520 varieties, while the HPA varieties had a significantly (p > 0.05) higher root length than the LPA and LS8520 varieties. No significant differences were found in the shoot length at the residual level of P application (Table 5). However, a significant difference (p < 0.05) was found at the optimum and higher levels of the P application rate. At the optimum level of P, the HPA varieties were found to have a higher shoot length than the LPA and SC701 varieties, while there were no significant differences between the HPA and SC701 varieties (Table 5). At a high level of P application, trends similar to the optimum P rate were observed. An increase in the P application rate was observed to increase the shoot length. There were non-significant differences (p > 0.05) within and across the treatment in the root: shoot ratio (Table 5).

### 3.3 Accelerated ageing vigour test

There were significant differences (p < 0.05) in the final germination, when the seeds were subjected to the AA test for 48 h and 72 h (Table 6). The germination percentage decreased by 14%, 14%, 16% and 20% in LPA, HPA, SC701 and LS8520, respectively, at the residual P fertilizer application rate, after 48 h of exposure to the AA test. At the optimum P application rate, it decreased by 13%, 7.7%, 13% and 14% in LPA, HPA, SC701 and LS8520, respectively, and at the high rate of P application it decreased by 7.8%, 8%, 9% and 5.4%, respectively in LPA, HPA, SC701 and LS8520. Significant differences were found in the final germination between the varieties at the residual level of the P application at 48 h of exposure to the AA test (Table 6). The final germination of LPA varieties was similar to that of the HPA and LS8520 varieties. However, at the optimum and higher levels of P application, there were no significant differences (p > 0.05) in the total germination within the treatments. The increase in the P application rate was observed to improve the final germination percentage in all the varieties. Furthermore, at 72 h of exposure to the AA test, the total germination of LPA varieties was similar (p > 0.05) to that of SC701 and LS8520 at the residual P level of application. In contrast, at the optimum and higher level of P application rates, there were no significant differences (p > 0.05) in the final germination within these treatments. There was an improved germination percentage with an increase in the P application rate.

**Table 6:** Final germination, Mean Germination Time (MGT) and Germination Velocity Index (GVI) of seed AA test exposed for 48 and 72 h under different P concentration.

Variety	P (mg/kg)	48h			72h		
		Final Germination (%)	MGT	GVI	Total germination (%)	MGT	GVI
LPA	18	78.0^ab^	3.38^ef^	22.0^ab^	74.0^ab^	4.27^e^	21.5^bc^
HPA		82.0^bc^	3.20^cd^	25.0^bc^	77.0^efd^	3.77^cd^	22.5^cd^
SC701		84.0^cde^	2.98^ab^	24.2^c^	79.0^bcdef^	3.86^de^	23.2^cde^
LS8520		75.0^a^	3.50^f^	20.2^a^	70.4^a^	3.84^d^	18.5^a^
LPA	26	83.0^bcd^	3.20^cd^	26.2^cd^	77.8^bcd^	3.65^cd^	23.4^cde^
HPA		87.3^cdef^	3.0^a^	27.0^cd^	78.0^bcde^	3.54^bcd^	23.4^cde^
SC701		87.0^cdef^	3.05^bc^	27.5^cd^	82.0^cdef^	3.12^ab^	24.4^de5^
LS8520		84.0^cde^	3.30^de^	25.7^cd^	76.0^abc^	3.42^abc^	19.9^ab^
LPA	34	89.0^ef^	2.90^ab^	28.2^cd^	84.6^ef^	3.02^a^	25.1^e^
HPA		88.0^def^	2.85^a^	27.9^cd^	83.0^def^	3.41^abc^	24.3^de^
SC701		90.0^f^	2.86^a^	28.9^d^	85.5^ef^	3.03^a^	25.6^e^
LS8520		87.6^cdef^	2.96^a^	28.8^d^	83.4^def^	3.18^ab^	23.8^cde^
LSD_(v×p)_		3.48	0.101	2.12	3.86	0.24	1.46
*P* (v)		<.001	<.001	<.001	<.001	<.001	<.001
(P)		<.001	<.001	<.001	<.001	<.001	<.001
(Pxv)		0.036	<.001	<.001	0.162	<.001	0.019
CV		2.9	2.3	5.6	3.4	4.8	4.5

Note: LPA = low phytic acid, HPA = high phytic acid, SC701 = white maize and LS8520 = yellow maize. Values within the same column sharing the same letter are not significant different at p < 0.05.

There were significant differences (p < 0.05) in the MGT when seeds were exposed to an AA vigor test for 48 and 72 h. In both instances, the MGT decreased with an increase in the application rate of P. However, the results indicated that the MGT increased by increasing the time of exposure from 48 h to 72 h. At the residual level, LPA had a higher MGT, but at the high rate of P application the differences were not significant (p > 0.05) between the varieties. There were no significant (p > 0.05) differences in the GVI observed between the varieties at both the optimum and high P fertilizer application rates. The differences were only observed at the residual P fertilizer application rate (Table 6), where SC701 was observed to have a higher GVI than other varieties.

## 4 Discussion

The objective of this study was to investigate the performance of low phytic acid maize populations, of tropical origin, to different levels of phosphorus application and to evaluate the seed quality, with respect to germination and vigour, of the seeds produced at different levels of fertilizer application. The results of this study showed a significantly lower plant height in the low phytic population at the residual (18 mg/kg) rate of P application, while at the optimum (26 mg/kg) and higher (34 mg/kg) levels, there were no significant differences (p > 0.05) in the plant height (Table 3). With the increase in the P application rate, the plant height of low phytic acid varieties also increased significantly, from the residual to the optimum rates of P application. On the other hand, the leaf numbers were found to be significantly lower (p < 0.05) than all the other varieties in the high phytic acid varieties at the residual level of P application (Table 3). With the increase in P application from 18 mg/kg to 26 mg/kg and 34 mg/kg, there were no significant differences in the plant leaf numbers within the treatments, except at 26 mg/kg, where the SC701 variety had significantly more leaves than all the other varieties. However, with the increase of the fertilizer application rate from 18 mg/kg to 26 mg/kg, there was no significant increase in the leaf numbers of all the varieties. The grain yield, on the other hand, increased with the increase in fertiliser application from 18 mg/kg to 26 mg/kg. However, at all levels of P application, the low phytic acid maize population produced a yield (p > 0.05) that was similar to the other varieties.

These results indicated a comparable and similar performance between the low and high phytic acid plants, with regards to their height and leaf numbers, although one commercial variety, SC701, outperformed all the other varieties. These results are, however, in contrast with what has been observed in low phytic acid mutants (*lpa 1-1*), where the yield was lower, when compared to normal cultivars (Naidoo et al., 2012). A similar performance between low and high phytic acid may indicate that the combination of low phytic acid synthetic populations may have produced a population that contains enough of the required reserves to produce acceptable yields. This also indicates the importance of applying the correct amount of fertilizer for achieving high yields. However, care must be taken when interpreting these results, since the variations beyond the studied seeds and location were not examined. The decline in days to the tasselling, silking and anthesis stages (Table 3), with the increase in P application, was observed in all varieties and this was in agreement with the work of Ali et al. (2019) and Ye et al. (2019). However, it contradicted the work of Amanullah (2015), who reported a delay in maize silking, due to increased P application. Liu et al. (2018) cited that the flowering time is genetically determined, but it can also be influenced by factors such as the soil fertility status, as well as the complex interaction of this with other environmental conditions.

The final germination percentage of the low phytic acid population was found to be comparable to other varieties at the 26 mg/kg and 34 mg/kg level of P fertilizer application, although it was observed to be significantly lower at 18 mg/kg than in other varieties (Table 5). With the increase in P application, however, the performance of low phytic acid populations improved significantly. Hrdličková et al. (2011) and El-Waraky (2014) also found that phosphorus application enhances seed germination. It has been found that, during seed germination, phosphorus stimulates early root formation and growth in plants. The results also showed no responses in the MGT, GVI and root:shoot ratio in all varieties, with the increased application of P. However, in the AA ageing vigor test, differences were found. At 48 h of exposure to the AA test, significant differences were found in the final germination between the varieties at the 18 mg/kg level of P application, with SC701 having the highest germination percentage (Table 6). The final germination of LPA varieties was similar (p > 0.05) to that of the HPA and LS8520 varieties. However, at the 26 mg/kg and 34 mg/kg of P application, there were no significant differences (p > 0.05) within the treatments in final germination. The increase in the P application rate was observed to improve the final germination percentage in all the varieties. These results indicate the complex interaction of the seed quality parameters that determine field performance. This suggests that different measures must be interpreted with care, when simulating the field performance of a particular seed-lot.

## 5 Conclusion

The results of this study indicated that the grain yield of low and high phytic acid synthetic populations was similar to that of commercial hybrids. This may suggest that the combination of low maize synthetic populations used in this study provided phosphorus reserves that were adequate to produce yields that are comparable to commercial hybrid varieties. The study also found that providing optimum P at planting improves the germination and vigor of the seedling during the early stages of crop development. Therefore, these low phytic acid synthetic populations may provide an alternative and a better solution than the low phytic acid mutants that have been released and that are not suited to the tropical and sub-tropical conditions of sub-Saharan Africa, where the problem of malnutrition still persists.

## Declarations

### Author contribution statement

Mohammed A.E. Bakhite: Conceived and designed the experiments; Analyzed and interpreted the data; Wrote the paper.

Nkanyiso J. Sithole; Khayelihle Ncama: Performed the experiments; Wrote the paper.

Lembe S. Magwaza: Contributed reagents, materials, analysis tools or data.

Alfred O. Odindo: Conceived and designed the experiments.

Shirly T. Magwaza: Performed the experiments.

### Funding statement

This research did not receive any specific grant from funding agencies in the public, commercial, or not-for-profit sectors.

### Data availability statement

Data will be made available on request.

### Declaration of interest statement

The authors declare no conflict of interest.

### Additional information

No additional information is available for this paper.
